# Dual decline in gait and cognition as a high-risk clinical phenotype: differential associations with cerebral amyloid-*β* deposition and the apolipoprotein E ε4 allele and implications for integrated assessment

**DOI:** 10.3389/fnagi.2026.1845747

**Published:** 2026-07-15

**Authors:** Chenxi Ren, Jiehua Zhu, Lin Huang, Tingjun Hu, Yihui Guan, Fang Xie, Jun Jin, Qihao Guo

**Affiliations:** 1 Department of Clinical Nutrition, Shanghai Sixth People's Hospital Affiliated to Shanghai Jiao Tong University School of Medicine, Shanghai, China; 2 Department of Gerontology, Shanghai Sixth People's Hospital, Shanghai Jiao Tong University School of Medicine, Shanghai, China; 3 Department of Nuclear Medicine & PET Center, Huashan Hospital, Fudan University, Shanghai, China

**Keywords:** aging, APOE ε4, cognitive decline, positive Aβ, slow gait

## Abstract

**Background:**

Gait slowing and cognitive impairment often coexist in older adults, yet their relationship with core Alzheimer’s disease (AD) biomarkers remains incompletely understood.

**Objective:**

To investigate the associations of isolated and combined slow gait (SG) and cognitive impairment subtypes with cerebral amyloid-*β* (Aβ) deposition and the apolipoprotein E ε4 (APOE ε4) allele in Chinese older adults.

**Methods:**

This cross-sectional study included 1,753 participants (mean age 65.9 years). Based on gait speed and cognitive status, participants were classified into six groups: normal, slow gait alone (SG-A), subjective cognitive decline alone (SCD-A), mild cognitive impairment alone (MCI-A), SCD with slow gait (SCD-SG), and MCI with slow gait (MCI-SG). 687 individuals underwent 18F-florbetapir positron emission tomography (PET) scans, 654 participants were examined for Apolipoprotein E (APOE) genotyping, and 618 participants had all relevant information recorded.

**Results:**

The MCI-SG group exhibited the most pronounced physical decline (slowest gait speed and weakest handgrip strength) and the highest burden of AD pathology, with a significantly higher prevalence of Aβ positivity (38%) and APOE ε4 carriage (32%) compared with other groups. While overall Aβ positivity rates across the six groups were not significantly different, logistic regression analyses revealed specific, strong associations. Aβ positivity was significantly associated with both SCD-SG (OR = 1.78, 95% CI: 1.03–3.08) and MCI-SG (OR = 1.85, 95% CI: 1.07–3.21) compared with the normal group. In contrast, APOE ε4 carriage was specifically and more strongly linked to MCI-SG (OR = 3.21, 95% CI: 1.41–7.31) compared with the SCD-A group. These combined gait-cognitive impairment phenotypes showed consistently stronger associations with AD biomarkers than isolated impairments across multiple reference groups. The risk was greatest for MCI-SG in individuals who were both Aβ positive and APOE ε4 carriers (OR = 2.27, 95% CI: 1.19–5.15).

**Conclusion:**

The co-occurrence of slow gait and mild cognitive impairment (MCI-SG) represents a distinct high-risk clinical phenotype strongly linked to AD pathology. Aβ and APOE ε4 show differential associations across the gait-cognitive spectrum. Integrated assessment of gait and cognition improves risk stratification in older adults and may guide early intervention strategies.

## Introduction

The global burden of dementia is rising sharply, with prevalence projected to increase from 57.4 million in 2019 to 152.8 million by 2050, underscoring an urgent need for early detection and intervention (GBD 2019 Dementia Forecasting Collaborators, 2022). While cognitive and motor declines are hallmark features of aging and neurodegenerative diseases, most research has focused on individuals aged 60 and above, leaving a critical evidence gap in younger populations, particularly among those aged 50–59 years (Maggio and Lauretani, 2019; Pike et al., 2022).

Slow gait, subjective cognitive decline (SCD), and mild cognitive impairment (MCI) are recognized as preclinical markers of Alzheimer’s disease (AD) and are associated with adverse outcomes such as disability, falls, and increased mortality (Maggio and Lauretani, 2019; Pike et al., 2022; Tian et al., 2021; Grande et al., 2019). Notably, gait slowing can emerge as early as midlife, suggesting it is not merely an age-related change but may reflect underlying neuropathology (Tian et al., 2021). Converging evidence indicates a strong link between slower gait speed and cognitive decline, with the combination of MCI and slow gait conferring a substantially higher risk of progression to dementia than either condition alone (Dumurgier et al., 2017; Doi et al., 2019; Peel et al., 2019; Doi et al., 2018; Doi et al., 2015). This interplay may be rooted in shared neural substrates, including prefrontal, parietal, and temporal regions, which govern both motor control and cognitive functions (Rosano et al., 2012; Annweiler et al., 2013; Rosso et al., 2013). Genetic studies further support common pathways, with certain variants, such as the APOE ε4 allele, implicated in both cognitive and motor deterioration (Holtzer et al., 2010; Hupfeld et al., 2018). Despite these insights, comprehensive studies characterizing cognitive profiles in individuals with slow gait—particularly across the continuum from SCD to MCI—remain scarce. This co-occurrence of subjective cognitive complaints and slow gait is recognized as Motoric Cognitive Risk (MCR) (Verghese et al., 2014), a predementia syndrome associated with increased risk of cognitive decline and dementia. In the present study, we further extend this concept by incorporating objective cognitive assessment and AD biomarkers, enabling a more refined characterization of the gait-cognitive spectrum.

The recent success of anti-amyloid monoclonal antibodies in AD trials has heightened the importance of early and accurate identification of individuals with amyloid-*β* (Aβ) pathology (Huang et al., 2024). Elevated Aβ burden, measured by positron emission tomography (PET), is associated with faster cognitive decline and slower gait even in cognitively normal older adults (van der Kall et al., 2021; Del Campo et al., 2016). Similarly, the APOE ε4 allele, the strongest genetic risk factor for sporadic AD, is linked to accelerated cognitive and motor decline (Sakurai et al., 2019). However, it is unclear how these key biomarkers—alone or in combination—relate to the co-occurrence of cognitive impairment and slow gait in community-dwelling adults, especially in those under 60.

To address these gaps, this study aimed to: (1) characterize and compare the clinical features of individuals with both cognitive decline and slow gait, specifically those with SCD plus slow gait (SCD-SG) and MCI plus slow gait (MCI-SG); (2) examine whether PET-Aβ positivity and the APOE ε4 allele are associated with these combined phenotypes in dementia-free Chinese adults aged 50 and above; and (3) describe the age-specific prevalence of SCD-SG and MCI-SG. We hypothesized that both Aβ positivity and APOE ε4 carriage would be independently and jointly associated with the co-occurrence of cognitive decline and slow gait. By elucidating these relationships, this study may offer new insights into the shared pathophysiology of motor and cognitive impairment and aid in the early identification of high-risk individuals in midlife and beyond.

## Methods

### Study population

This is a retrospective study. Between January 2019 and June 2023, a total of 3,034 individuals participated in a study conducted by the Department of Geriatrics at Shanghai Sixth People’s Hospital. All participants underwent neuropsychological assessments performed by trained personnel in a designated evaluation room (Supplementary Figure S1). An 18F-florbetapir PET scan was conducted within 3 months following blood sample collection. We excluded individuals based on the following criteria: (1) those under 50 years old (*n* = 303); (2) those who met the American Psychiatric Association’s (APA) diagnostic criteria for dementia as outlined in the 4th edition of the Diagnostic and Statistical Manual of Mental Disorders (*n* = 645); (3) individuals with a history of major psychological disorders (*n* = 32); and (4) those with disabilities or lacking records on gait speed (*n* = 301). Ultimately, 1,753 participants were included in the analysis, with 687 undergoing the 18F-florbetapir PET scan, 654 providing blood samples for APOE genotyping, and 618 having both PET scan and APOE genotyping data recorded. To evaluate the potential confounding effect of cerebrovascular pathology, we performed a sensitivity analysis excluding participants with a history of cerebral infarction (*n* = 81). All primary analyses were repeated, and the results were consistent with those from the full sample, supporting the robustness of our finding. The study received approval from the Ethics Committee of Shanghai Sixth People’s Hospital (approval number 2019-041), and all participants provided written informed consent in accordance with the principles of the Helsinki Declaration.

### Measurements at baseline

Basic patient information regarding chronic illnesses, medical history, and lifestyle factors—such as age, gender, smoking and drinking habits, heart disease, hypertension, type 2 diabetes, osteoporosis, cerebral infarction, fractures, and liver cirrhosis—was verified through medical record reviews conducted by qualified physicians. Heart disease encompassed a history of coronary artery disease and arrhythmias. Cerebral infarction was validated as a history of ischemic attacks confirmed by cerebral computer tomography (CT) or magnetic resonance imaging (MRI) scans. Handgrip strength was measured in kilograms using a dynamometer (WCS-100, Nantong, China).

The SCD group encompassed individuals who reported a perceived decline in cognitive abilities despite the absence of objective evidence as determined by neuropsychological assessments. Additionally, this group included those whose cognitive changes can be identified during the preclinical stage of AD through the application of sensitive neuropsychological measures (Thomas et al., 2018; Jessen et al., 2014). The inclusion criteria for MCI were adapted from the guidelines established by Petersen et al. and consist of the following: (1) The presence of cognitive concerns or complaints reported by the individual, an informant, a nurse, or a physician within the past year; (2) Maintenance of normal cognitive function as assessed by the Montreal Cognitive Assessment Basic version (MoCA-BC) (Chen et al., 2016), with scores indicating normalcy based on educational attainment (primary school: 19–14, secondary school: 22–16, university and above: 24–17); (3) Evidence of objective cognitive impairment as indicated by standard neuropsychological tests, with scores falling at least 1.5 standard deviations (SD) below the normative data for age and education; (4) Preservation of activities of daily living, with only minor impairments in instrumental activities of daily living, specifically, no more than one item from the Chinese version of the Activities of Daily Living Scale (ADLs) exhibited significant changes, or the total score was less than 26 (Chen et al., 1995); (5) The absence of dementia (Petersen, 2024; Jack et al., 2018). Participants classified as SCD-SG exhibited both subjective cognitive decline and reduced gait speed, while those categorized as MCI-SG demonstrated mild cognitive impairment alongside slow gait speed.

Gait speed was assessed utilizing the Timed Up and Go test, which encompasses a 6-meter distance. The criteria for categorizing slow gait speed exhibit variability across different studies. In our investigation, we undertook a comprehensive analysis of various age cohorts. Slow gait speed was operationally defined as a walking speed that falls below the age-specific and sex-specific (male or female) mean walking speeds within our sample (Dreyer-Alster et al., 2022). Specifically, slow gait speed was characterized as less than 0.70 m/s, 0.71 m/s, 0.65 m/s, and 0.62 m/s for males in the age groups of 50–59 years, 60–64 years, 65–69 years, and ≥70 years, respectively; and as less than 0.71 m/s, 0.70 m/s, 0.67 m/s, and 0.60 m/s for females in the corresponding age categories.

### Amyloid PET imaging

The 18F-florbetapir PET scan was conducted to evaluate the Aβ burden in the brain using a PET/CT system (Biograph mCT Flow PET/CT, Siemens, Erlangen, Germany) (Ren et al., 2023). The identification of positive 18F-florbetapir PET images was carried out through visual rating, adhering to established guidelines for the interpretation of amyloid PET scans (Lundeen et al., 2018). The interpretation of the amyloid PET images was executed independently by three specialists in nuclear medicine, with the final results contingent upon the consensus of at least two of the specialists.

### APOE ε4 genotyping

Blood samples were obtained through venipuncture. Additionally, APOE genotyping was conducted utilizing polymerase chain reaction (PCR) methods, with participants categorized as APOE ε4 carriers if they possessed at least one ε4 allele.

### Statistical analysis

Statistical analyses were conducted using SAS version 9.4 (SAS Institute, Cary, NC). Continuous variables, including age, education, handgrip strength, gait speed, and neuropsychological test scores, are presented as means with SD. Categorical variables, such as sex, smoking status, alcohol consumption, chronic diseases, and the presence of positive Aβ and APOE ε4 carriers, are reported as counts and percentages. To assess differences among groups for continuous variables, one-way analysis of variance (ANOVA) was employed. For categorical variables, the chi-square test was utilized to evaluate group differences. A column scale test with Bonferroni correction (*z*-test) was applied to identify significant differences across groups. Multivariate logistic regression analysis was performed to ascertain the factors influencing MCI-SG and SCD-SG. A significance level was set at a *p*-value of 0.05. Graphical representations were generated using GraphPad Prism version 9.

## Results

### Participant characteristics and group overview

**Figure 1:**
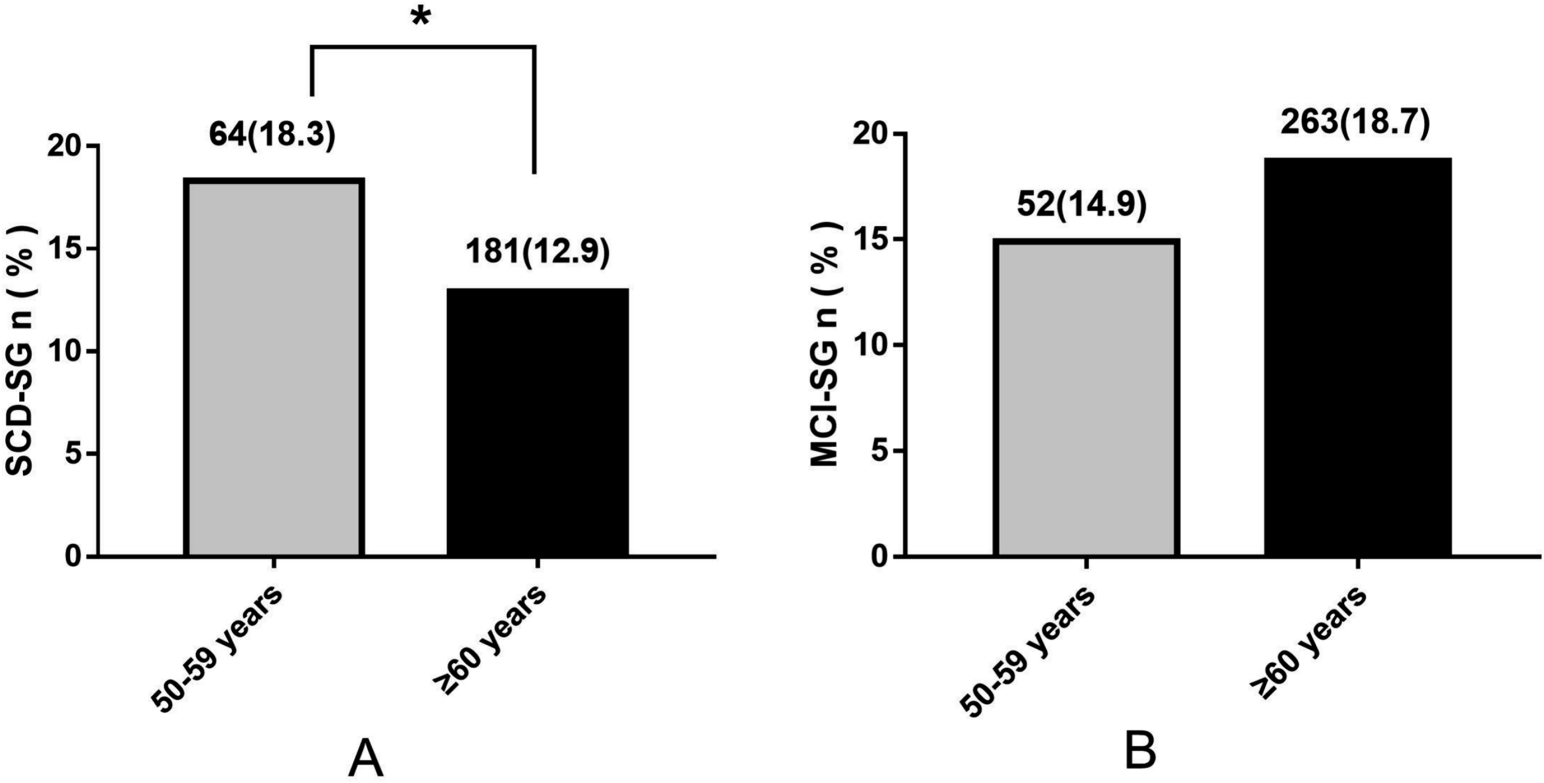
**(A)** The prevalence of SCD-SG was 18.3% in aged 50–60 years group. The prevalence of SCD-SG was 12.9% in aged 50–60 years group in above 60 years group. There is significant difference between two groups. This figure showed the age-related distribution of the two most clinically relevant phenotypes, highlighting the disproportionately high prevalence of SCD-SG in the 50–59 year old group, which supports the subsequent discussion of an early intervention window. **(B)** The prevalence of MCI-SG was 14.9% in aged 50–60 years group. The prevalence of SCD-SG was 18.7% in aged 50–60 years group in above 60 years group. There is no difference between two groups.

A total of 1,753 individuals participated in this study, comprising 571 men and 1,182 women, with a mean age of 65.9 years. Among the participants, 335 (19.1%) exhibited slow gait alone (SG-A), 221 (12.0%) were identified with SCD alone (SCD-A), 201 (11.5%) were diagnosed with MCI alone (MCI-A), 245 (13.4%) presented with SCD-SG, and 315 (17.8%) were classified as MCI-SG (Table 1). Compared with individuals older than 60 years, individuals aged 50 to 60 years were particularly susceptible to developing SCD-SG (18.3%) (Figure 1).

**Table 1:** Baseline characteristics of participants in six groups.

Characteristics	Normal (*n* = 446)	SG-A (*n* = 335)	SCD-A (*n* = 211)	MCI-A (*n* = 201)	SCD-SG (*n* = 245)	MCI-SG (*n* = 315)	*p*
Demographics							
Age (years)	64.7 ± 7.4^b^^,^^c^^,^^d^^,^^f^	65.8 ± 7.5^a^^,^^d^^,^^f^	66.2 ± 7.0^a^	67.3 ± 6.6^a^^,^^b^^,^^e^	64.9 ± 7.7^d^^,^^f^	67.2 ± 7.1^a^^,^^b^^,^^e^	<0.001
Male *n* (%)	156 (35.0)	108 (32.2)	68 (32.2)	66 (32.8)	64 (26.1)	109 (34.6)	0.376
Smoking *n* (%)	50 (20.7)	41 (24.9)	20 (18.0)	27 (23.3)	20 (16.8)	46 (26.0)	0.605
Drinking *n* (%)	73 (17.4)	56 (17.4)	34 (17.0)	33 (17.1)	43 (18.2)	56 (18.5)	0.668
Education (years)	12.5 ± 3.2^d^^,^^e^^,^^f^	12.4 ± 3.1^d^^,^^e^^,^^f^	12.0 ± 3.3^d^^,^^f^	11.0 ± 3.4^a^^,^^b^^,^^c^^,^^e^	11.8 ± 3.3^a^^,^^b^^,^^d^^,^^e^	10.7 ± 3.3^a^^,^^b^^,^^c^^,^^e^	<0.001
Global Aβ deposition (SUVr)	1.25 ± 0.11	1.24 ± 0.14f	1.25 ± 0.09	1.28 ± 0.13	1.25 ± 0.15	1.25 ± 0.15	0.665
APOEε4 carrier	30 (19.4)^f^	21 (15.8)^f^	10 (13.7)^f^	15 (24.6)	21 (19.6)^f^	40 (32.0)^a^^,^^b^^,^^c^^,^^e^	0.007
MoCA-BC total scores	25.7 ± 2.8^b^^,^^c^^,^^d^^,^^e^^,^^f^	25.1 ± 2.9^a^^,^^c^^,^^d^^,^^e^^,^^f^	24.1 ± 3.3^a^^,^^b^^,^^d^^,^^f^	21.8 ± 3.4^a^^,^^b^^,^^c^^,^^e^^,^^f^	23.7 ± 3.8^a^^,^^b^^,^^d^^,^^f^	20.6 ± 3.7^a^^,^^b^^,^^c^^,^^d^^,^^e^	<0.001
Gait speed (m/s)	0.79 ± 0.12^b^^,^^d^^,^^e^^,^^f^	0.57 ± 0.10^a^^,^^c^^,^^d^^,^^f^	0.78 ± 0.11^b^^,^^d^^,^^e^^,^^f^	0.75 ± 0.11^a^^,^^b^^,^^d^^,^^e^^,^^f^	0.56 ± 0.09^a^^,^^c^^,^^d^^,^^f^	0.53 ± 0.10^a^^,^^b^^,^^c^^,^^d^^,^^e^	<0.001
Handgrip strength (kg)	24.8 ± 7.6^b^^,^^e^^,^^f^	23.6 ± 8.2^a^^,^^c^^,^^e^^,^^f^	25.1 ± 10.9^b^^,^^e^^,^^f^	23.5 ± 7.9^f^	22.0 ± 7.6^a^^,^^b^^,^^c^	21.4 ± 7.2^a^^,^^b^^,^^c^^,^^d^	<0.001
Heart disease *n* (%)	25 (5.61)	26 (7.76)	28 (13.27)	18 (8.96)	27 (11.02)	35 (11.11)	0.004
Hypertension *n* (%)	152 (34.08)	115 (34.33)	75 (35.55)	76 (37.81)	90 (36.73)	123 (39.05)	0.115
Type 2 diabetes *n* (%)	50 (11.21)	46 (13.73)	30 (14.22)	22 (10.95)	46 (18.78)	53 (16.83)	0.010
Osteoporosis *n* (%)	38 (8.52)	29 (8.66)	25 (11.85)	19 (9.45)	38 (15.51)	40 (12.70)	0.006
Cerebral infarction *n* (%)	14 (3.14)	19 (5.67)	7 (3.32)	5 (2.49)	9 (3.67)	27 (8.57)	0.020
Fracture *n* (%)	33 (7.40)	30 (8.96)	26 (12.32)	24 (11.94)	30 (12.24)	40 (12.70)	0.006
Liver cirrhosis *n* (%)	9 (2.02)	4 (1.19)	6 (2.84)	2 (1.00)	14 (5.71)	16 (5.08)	0.001

SG-A, slow gait alone; SCD-A,subjective cognitive decline alone; MCI-A, mild cognitive impairment alone; SCD-SG, SCD plus slow gait; MCI-SG, MCI plus slow gait.

a Significantly different from the normal group.

b Significantly different from the slow gait group.

c Significantly different from the SCD-A group.

d Significantly different from the MCI-A group.

e Significantly different from the SCD-SG group.

f Significantly different from the MCI-SG group.

^*^ Significantly different between male and female.

### Chronic conditions, Aβ positivity, and APOE ε4 carrier status across groups

The analysis revealed no significant differences in sex, smoking, drinking, prevalence of hypertension across the six groups (Table 1). Participants diagnosed with MCI-SG were older, with a mean age of 67.2 ± 7.1 years, and had a lower educational level (10.7 ± 3.3 years). Notably, individuals in the MCI-SG group demonstrated the slowest walking speeds (0.53 ± 0.10 m/s) and the lowest handgrip strength (20.6 ± 3.7 kg) when compared to the other five groups. Conversely, participants with SCD-SG exhibited higher walking speeds (0.56 ± 0.09 m/s) and greater handgrip strength (22.0 ± 7.6 kg) than those with MCI-SG, although they were still lower than those of the normal, SG-A, SCD-A, and MCI-A groups. Additionally, differences in walking speed and handgrip strength were observed between males and females in certain groups. Our findings revealed that SCD-SG and MCI-SG were significantly associated with a higher prevalence of chronic conditions, including cardiovascular disease, type 2 diabetes, osteoporosis, cerebral infarction, fractures, and liver cirrhosis. However, due to the cross-sectional nature of this study, the direction of these associations cannot be determined and longitudinal studies are warranted to clarify the temporal relationship. Importantly, the prevalence of hypertension did not differ among the six groups. No significant differences in global amyloid-beta (Aβ) deposition (measured by Standardized Uptake Value ratio, SUVr) were found across the six groups. The prevalence of APOE ε4 carriers in MCI-A and MCI-SG were notably higher compared to the other groups (Table 1).

### Association of Aβ and APOE ε4 with SCD-SG and MCI-SG

**Figure 2:**
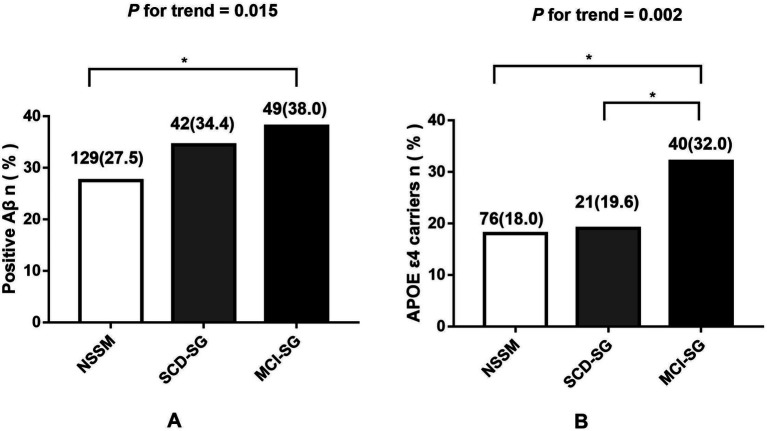
**(A)** The prevalence of positive Aβ was 27.5, 34.4 and 38.0% in NSSM, SCD-SG and MCI-SG. The prevalence of positive Aβ was higher in MCI-SG than NSSM. A progressive increase in the prevalence of positive Aβ across the NSSM, SCD-SG, and MCI-SG groups (*P* for trend = 0.015). **(B)** The prevalence of APOEε4 + was 18.0, 19.6 and 32.0% in NSSM, SCD-SG and MCI-SG. The prevalence of APOEε4 + was higher in MCI-SG than SCD-SG and NSSM. A progressive increase in the prevalence of APOE ε4 carriers across the NSSM, SCD-SG, and MCI-SG groups (*P* for trend = 0.002).

We created an NSSM group comprising the normal, SG-A, SCD-A, and MCI-A subgroups, forming the acronym from their first letters. The prevalence of positive Aβ and the presence of the APOE ε4 allele exhibited statistically significant differences among NSSM group (normal, SG-A, SCD-A, and MCI-A), SCD-SG and MCI-SG group. Specifically, the prevalence of positive Aβ was found to be 38% in the MCI-SG group, which is higher than that observed in the NSSM group. Additionally, the prevalence of APOE ε4 carriers was 32% in the MCI-SG group, surpassing the rates found in both the SCD-SG and NSSM group. These findings indicated a progressive increase in the prevalence of positive Aβ and APOE ε4 carriers across the NSSM, SCD-SG, and MCI-SG groups (Figure 2).

**Figure 3:**
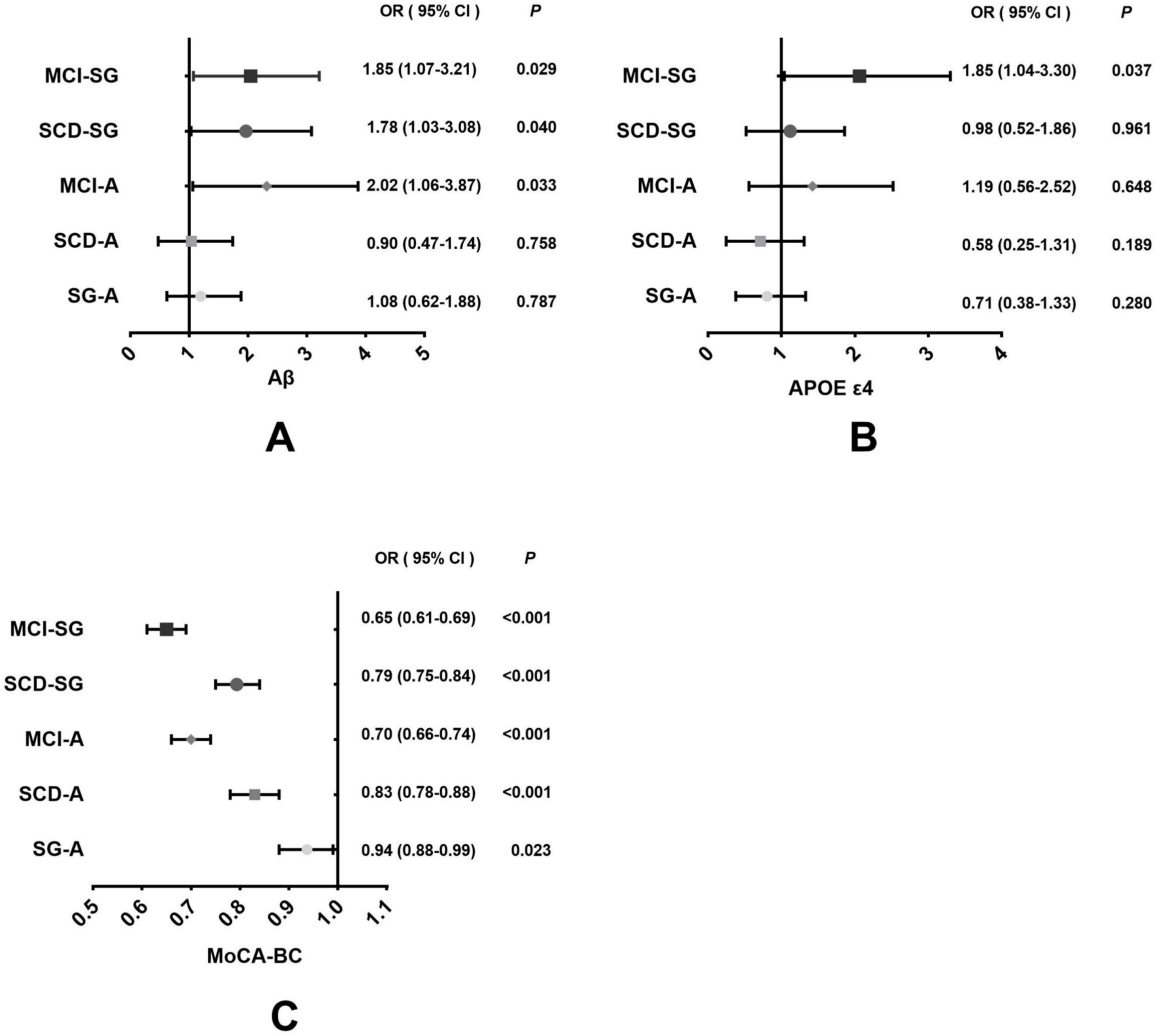
With the normal group as a reference, after adjusting for confounding factors, the association of MCI-SG with positive Aβ and APOE ε4 carrier remained significant difference (OR = 1.85, 95% CI: 1.07–3.21; OR = 1.85, 95% CI: 1.04–3.30); SCD-SG was significantly associated with positive Aβ (OR = 1.78, 95% CI: 1.03–3.08), but there was no difference with APOE ε4 carrier **(A,B)**; the risk of MCI-A was increased in positive Aβ **(A)**. There was no difference between positive Aβ, APOE ε4 carrier and SG-A **(A,B)**. SG-A, SCD-A, MCI-A, SCD-SG and MCI-SG were all associated with MoCA-BC **(C)**.

With the normal group as a reference, after adjusting for confounding factors, the association of MCI-SG with positive Aβ and APOE ε4 carrier remained significant [odd ratio (OR) = 1.85, 95% confidence interval (CI): 1.07–3.21; OR = 1.85, 95% CI: 1.04–3.30]; SCD-SG was significantly associated with positive Aβ (OR = 1.78, 95% CI: 1.03–3.08), but there was no difference with APOE ε4 carrier (Figures 3A,B); the risk of MCI-A was increased in positive Aβ (Figure 3A). There was no difference between positive Aβ, APOE ε4 carrier and SG-A (Figures 3A,B). SG-A, SCD-A, MCI-A, SCD-SG and MCI-SG were all associated with MoCA-BC (Figure 3C).

Above all, after controlling for age, sex, education, handgrip strength, heart disease, type 2 diabetes, osteoporosis, cerebral infarction, fracture, and liver cirrhosis, we also examined the risk of SCD-SG and MCI-SG in positive Aβ and APOE ε4 carriers using the SG-A, SCD-A, MCI-A, and NSSM groups as references, respectively. Compared to SG-A, the risk of MCI-SG was increased in APOE ε4 carriers (OR = 2.62, 95% CI: 1.40–4.91). In comparison to SCD-A, individuals with positive Aβ had a higher risk of SCD-SG or MCI-SG (OR = 1.97, 95% CI: 1.01–3.85; OR = 2.05, 95% CI: 1.05–4.00); APOE ε4 carriers had a higher risk of MCI-SG (OR = 3.21, 95% CI: 1.41–7.31). Positive Aβ or APOE ε4 carriers had a higher risk of MCI-SG (OR = 1.59, 95% CI: 1.01–2.48; OR = 2.16, 95% CI: 1.34–3.48) when compared with NSSM (Table 2).

**Table 2:** OR and 95% (CI) for the associations of positive Aβ and APOE ε4 carrier with risk of SCD-SG and MCI-SG.

Characteristics	SCD-SG	MCI-SG
	OR (95% CI)	OR (95% CI)
Positive Aβ		
SG-A (reference)	1.65 (0.94–2.89)	1.71 (0.98–3.00)
SCD-A (reference)	1.97 (1.01–3.85)^*^	2.05 (1.05–4.00)^*^
MCI-A (reference)	0.88 (0.46–1.69)	0.91 (0.48–1.73)
NSSM (reference)	1.55 (0.99–2.44)	1.59 (1.01–2.48)^*^
APOE ε4 carrier		
SG-A (reference)	1.39 (0.70–2.77)	2.62 (1.40–4.91)^*^
SCD-A (reference)	1.71 (0.72–4.05)	3.21 (1.41–7.31)^*^
MCI-A (reference)	0.83 (0.37–1.84)	1.55 (0.75–3.24)
NSSM (reference)	1.15 (0.66–2.01)	2.16 (1.34–3.48)^*^

The model adjusted for age, sex, education, handgrip strength, heart disease, type 2 diabetes, osteoporosis, cerebral infarction, fracture, liver cirrhosis. *p < 0.05.

Moreover, the risk of MCI-SG in positive Aβ/APOE ε4 carriers was 2.27 times higher than that of negative Aβ/APOE ε4 nocarrier, and the risk of MCI-SG in negative Aβ/APOE ε4 carriers was 2.45 times higher than that of negative Aβ/APOE ε4 carriers when compared to NSSM (Table 3).

**Table 3:** OR and 95% (CI) for the associations of positive Aβ/ APOE ε4 nocarrier, negative Aβ/APOE ε4 carrier and positive Aβ/APOE ε4 carrier with risk of SCD-SG and MCI-SG.

Characteristics	SCD-SG	MCI-SG
	OR (95% CI)	OR (95% CI)
Positive Aβ/APOE ε4 nocarrier vs. negative Aβ/APOE ε4 nocarrier		
Normal (reference)	1.92 (0.98–3.79)	2.01 (1.04–4.21)^*^
SG-A (reference)	1.45 (0.74–2.83)	1.58 (0.80–3.13)
SCD-A (reference)	1.62 (0.73–3.60)	1.76 (0.78–3.99)
MCI-A (reference)	0.93 (0.41–2.08)	1.01 (0.45–2.26)
NSSM (reference)	1.51 (0.87–2.63)	1.62 (0.92–2.87)
Negative Aβ/APOE ε4 carrier vs. negative Aβ/APOE ε4 nocarrier		
Normal (reference)	0.83 (0.32–2.12)	2.54 (1.17–5.54)^*^
SG-A (reference)	0.90 (0.34–2.38)	2.75 (1.22–6.21)^*^
SCD-A (reference)	0.72 (0.25–2.13)	2.22 (0.87–5.68)
MCI-A (reference)	0.61 (0.19–1.93)	1.88 (0.69–5.11)
NSSM (reference)	0.80 (0.35–1.83)	2.45 (1.30–4.60)^*^
Positive Aβ/APOE ε4 carrier vs. negative Aβ/APOE ε4 nocarrier		
Normal (reference)	1.48 (0.62–3.56)	2.00 (0.84–4.74)
SG-A (reference)	2.27 (0.85–6.05)	3.06 (1.16–8.04)^*^
SCD-A (reference)	11.60 (1.41–95.81)^*^	15.66 (1.90–129.02)^*^
MCI-A (reference)	0.91 (0.31–2.72)	1.23 (0.43–3.53)
NSSM (reference)	1.89 (0.89–4.02)	2.27 (1.19–5.15)^*^

Negative Aβ/APOE ε4 nocarrier, individuals with negative Aβ and APOE ε4 nocarrier; positive Aβ/APOE ε4 nocarrier, individuals with positive Aβ and APOE ε4 nocarrier; negative Aβ/APOE ε4 carrier, individuals with negative Aβ and APOE ε4 carrier; positive Aβ/APOE ε4 carrier, individuals with positive Aβ and APOE ε4 carrier.

The model adjusted for age, sex, education, handgrip strength, heart disease, type 2 diabetes, osteoporosis, cerebral infarction, fracture, liver disease. *p < 0.05.

### Sensitivity analysis

To evaluate the potential confounding effect of cerebrovascular pathology, we repeated all primary analyses after excluding participants with a self-reported history of cerebral infarction (*n* = 1,672). The associations between MCI-SG and Aβ positivity (OR = 2.05, 95% CI: 1.16–3.60, *p* = 0.013) and APOE ε4 carriage (OR = 1.83, 95% CI: 1.01–3.31, *p* = 0.045) remained significant, with effect sizes consistent with those from the full sample. Other key findings, including the association between Aβ and SCD-SG, were also essentially unchanged. These results confirm the robustness of our primary conclusions (detailed results are provided in Supplementary Tables S1–S4).

## Discussion

This cross-sectional study elucidated the complex interrelationship between gait speed, cognitive status, and AD biomarkers in a community-based older population. The study incorporated both Aβ-PET imaging and APOE ε4 genotype to analyze their independent and synergistic effects on cognitive and motor functions. By stratifying participants into distinct phenotypic groups, we identified MCI-SG as a clinically distinct and high-risk subgroup, characterized by a unique profile of physiological frailty and AD pathology.

MCI-SG is a high-risk phenotype with aggregated pathophysiological burden. Our data robustly indicate that the co-occurrence of objectively measured slow gait and MCI is not merely an additive effect but represents a synergistic, high-risk clinical entity. The MCI-SG group exhibited the most pronounced physical decline (lowest gait speed and handgrip strength) and the highest prevalence of both Aβ positivity (38%) and APOE ε4 carriage (32%). Critically, these associations remained significant after adjusting for a comprehensive set of demographic and health confounders. Such a convergence of motor and cognitive deficits, alongside an elevated AD biomarker load, strongly suggests that MCI-SG is a phenotype particularly enmeshed in the neurodegenerative process, potentially reflecting more advanced or diffuse brain pathology affecting both cortical and subcortical circuits. The coexistence of gait impairment and cognitive decline is not only an aging phenomenon, but also involves overlapping pathological events in the subcortical circuits and executive functions of the frontal lobe (Xiang et al., 2022). Therefore, a clear understanding of the fundamental causes, biology, and mechanisms of the different early stages of AD-related pathology may contribute to early identification of high-risk dementia (Xiang et al., 2022). Although the overall comparison of Aβ positivity rates across the six groups did not reach statistical significance, this finding suggests that in older adults, Aβ pathology may represent a continuous spectrum, and the comorbid phenotype serves as a robust clinical integrator that can effectively identify high-risk subgroups with greater pathological burden, demonstrating superior value over the assessment of isolated symptoms.

We showed differential roles of Aβ and APOE ε4 in the gait-cognitive spectrum. A key novel insight is the differential association of core AD biomarkers with specific phenotypic combinations. We found that Aβ positivity was significantly associated with both SCD-SG and MCI-SG, whereas APOE ε4 carriage was predominantly and strongly linked to MCI-SG. This pattern suggests a temporal or mechanistic hierarchy: Aβ pathology may be a common upstream contributor to the early nexus of subjective concern and motor slowing, while the APOE ε4 allele appears to exert a more potent or specific effect in driving the progression to, or coexistence with, overt objective cognitive impairment (MCI). Furthermore, the finding that APOE ε4 carriers without Aβ positivity still had a 2.45-fold higher risk of MCI-SG implies that this genetic risk factor may promote the phenotype through non-Aβ pathways, such as tauopathy, cerebrovascular disease, or impaired neuronal resilience. This broadens our understanding of the mechanisms by which APOE ε4 contributes to neurodegeneration, highlighting its multifaceted role beyond amyloid-beta deposition. Some studies showed that deposition of cerebral Aβ (mainly in the frontal, striatal, temporal, parietal, anterior cingulate, precuneus, and occipital cortices) could lead to cognitive impairment resulting in weakness and slow gait speed, and that the connectivity of Aβ was associated with executive function capabilities and APOE ε4 carrier status, which regulated cortical control of gait and tau hyperphosphorylation (Nadkarni et al., 2017; Shen et al., 2020; Verghese et al., 2011). Furthermore, regardless of the APOE status, a decreased gait speed was related to Aβ deposition in the posterior and anterior putamen (which shared a direct interaction with the pre-motor cortex, anterior cingulate, and sensorimotor cortex) and occipital cortex (linked with the visual cortex) (Del Campo et al., 2016; Semba et al., 2020). However, there was another viewpoint that APOE ε4 increased Aβ deposition in preclinical AD, which influenced cortical control of gait by both Aβ-related and unrelated processes, and this was supported by Neelesh K. Nadkarni et al.’s finding that gait speed was modestly associated with Aβ deposition independent of cardiac risk, hippocampal volume, and small-vessel disease burden, and that this relationship was attenuated by APOE ε4 and cognition in older adults without dementia (Nadkarni et al., 2017). Furthermore, a recent clinico-pathological study in Parkinson’s disease (PD) demonstrated that cognitive impairment and AD neuropathological changes were associated with worse gait and freezing of gait, reinforcing the hypothesis that both cognitive decline and AD co-pathology exert a dual influence on motor function across different neurodegenerative conditions (Parmera et al., 2025).

Beyond AD co-pathology, other age-related pathologies—including tauopathy, cerebrovascular disease (CVD), and *α*-synuclein deposition—can independently or synergistically contribute to gait impairment in older adults. In community-dwelling older adults, macroscopic infarcts (particularly subcortical) and atherosclerosis exert separate and synergistic effects with Lewy body pathology to potentiate parkinsonian gait (Agrawal et al., 2023). Longitudinal-multicohort analyses further reveal that motor decline often precedes cognitive decline, and the timing of pathological associations varies: tau tangles are significantly associated with gait decline beginning approximately 3.49 years before death, whereas macroinfarcts are associated with gait decline starting around 9.25 years before death—earlier than their association with cognitive decline (6.65 years before death) (Oveisgharan et al., 2024). These findings underscore the importance of considering mixed pathologies, particularly CVD and α-synuclein deposition, in understanding and managing gait impairment in aging populations.

Additionally, our research showed that a low level of MoCA-BC was associated with a higher risk of slow gait. This suggests that cognitive function may be enhanced by appropriate exercise and walking speed acceleration, which could reverse brain tissue loss and prevent AD. James A. Mortimer et al. showed those who walked faster had improved scores on some cognitive tests compared to slower walkers, and fast walkers experienced slightly less brain tissue loss than slow walkers (Mortimer et al., 2012).

In Chinese adults over 50, we also characterized, for the first time, the age-specific distributions of gait speed, cognitive function, and the co-occurrence of slow gait and cognitive decline. We ought to focus more on their subjective cognitive perception and walking speed in those aged 50–60 years. We speculate that the observed phenomenon is due to a severe decline in both walking speed and cognitive function with age (Sathyan et al., 2023). The incidence of slow gait accompanied by subjective cognitive complaints increases as people grow older (Xiang et al., 2022). We have discussed that cognitive frailty in Chinese aged ≥50 years increases risk of ≥3 chronic diseases, with the SCD-SG group at highest risk, highlighting the need for attention to cognitive frailty and multimorbidity (Zhu et al., 2025).

Several limitations must be acknowledged. The cross-sectional design precludes causal inferences about the temporal sequence of gait decline, cognitive impairment, and biomarker accumulation. Longitudinal studies are essential to determine whether slow gait precedes, follows, or co-evolves with cognitive and pathological changes. Our sample was drawn from a specific demographic; generalizability to other ethnic and younger populations requires validation. Furthermore, gait was assessed as speed alone; incorporating measures of gait variability, rhythm, and dual-task cost may yield even finer phenotypic discrimination.

## Conclusion

The study demonstrates that the co-occurrence of slow gait and cognitive impairment, particularly the MCI-SG phenotype, identifies a distinct subgroup of older adults with the greatest burden of physical decline and AD pathology. Our findings reveal a differential pattern of association: cerebral Aβ deposition is linked to earlier combined phenotypes (SCD-SG and MCI-SG), while the APOE ε4 allele shows a specific and stronger association with the more advanced MCI-SG stage. Critically, integrated assessment of gait and cognition provides superior risk stratification for underlying AD pathology compared to evaluating either domain in isolation. The MCI-SG phenotype emerges as a high-risk clinical entity that warrants priority for biomarker confirmation and potential early intervention. These results underscore the importance of incorporating simple gait speed measurements into cognitive evaluations in community and clinical settings to enhance the early identification of individuals at elevated risk for AD (Supplementary Figure S2).

## Data Availability

The original contributions presented in the study are included in the article/Supplementary material, further inquiries can be directed to the corresponding author.
