# Supplementary material for: Dual decline in gait and cognition as a high-risk clinical phenotype: differential associations with cerebral amyloid-β deposition and the apolipoprotein E ε4 allele and implications for integrated assessment
Source: Front Aging Neurosci. 2026 Jul 15;18:1845747. doi: 10.3389/fnagi.2026.1845747 (PMC13415942; doi:10.3389/fnagi.2026.1845747)

**Supplemental Figure1: Flowchart for subjects enrolled in this study.**


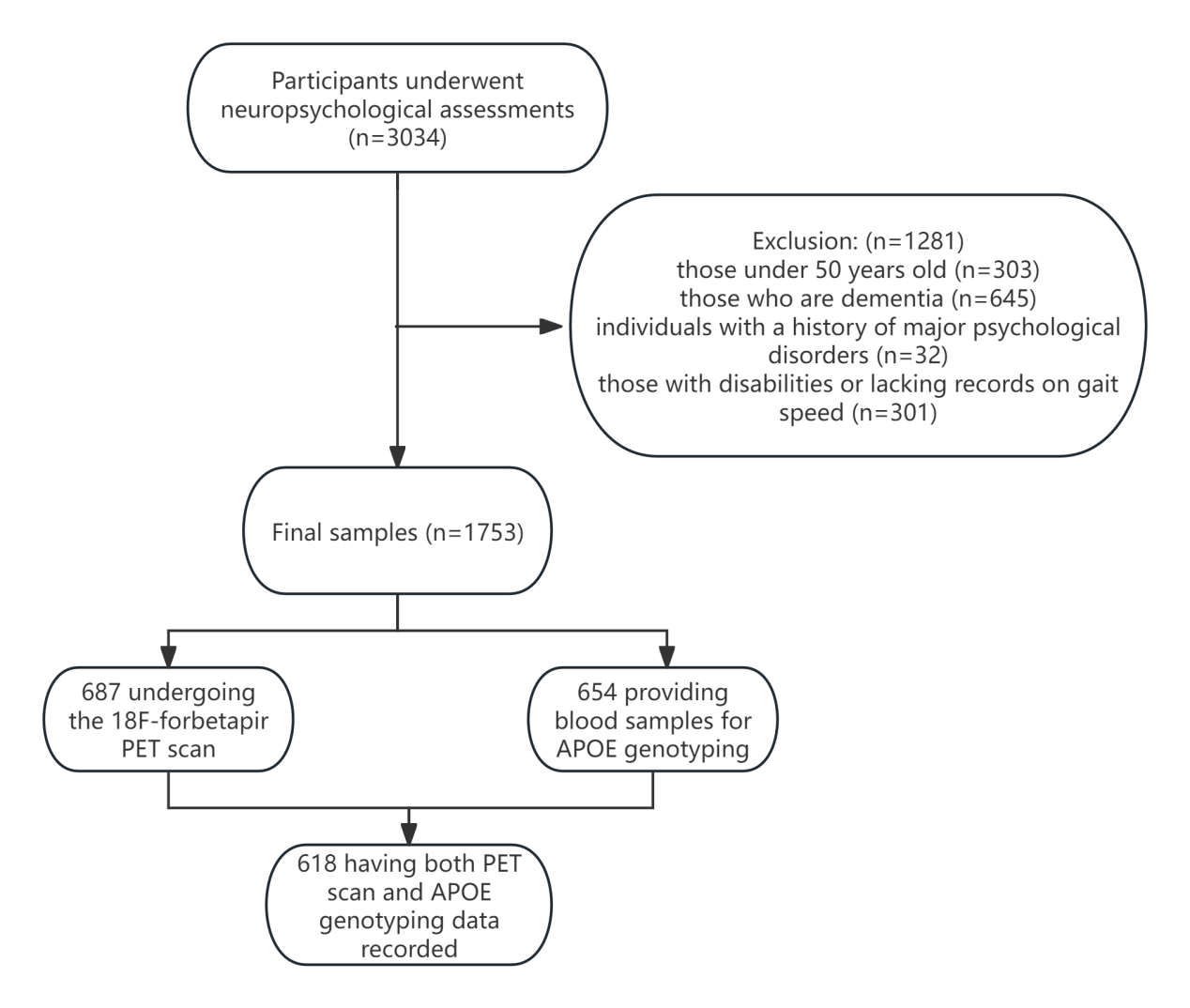


**Supplemental Figure2: Research Workflow Diagram.**


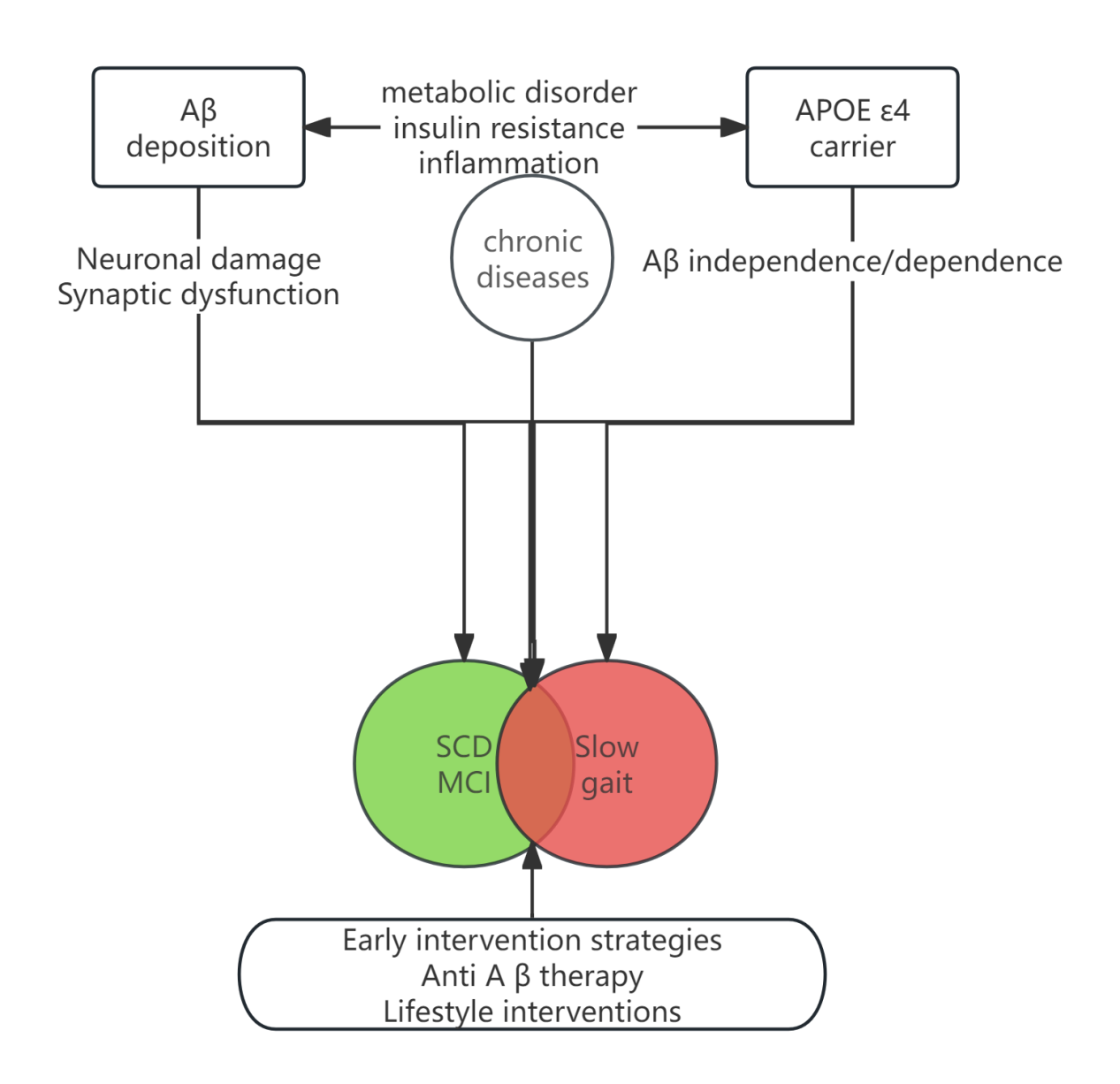

Supplement: Supplementary file 1 [file Data_Sheet_1.DOC]
