# Supplementary material for: Dual decline in gait and cognition as a high-risk clinical phenotype: differential associations with cerebral amyloid-β deposition and the apolipoprotein E ε4 allele and implications for integrated assessment
Source: Front Aging Neurosci. 2026 Jul 15;18:1845747. doi: 10.3389/fnagi.2026.1845747 (PMC13415942; doi:10.3389/fnagi.2026.1845747)
Supplement: Supplementary file 2 [file Data_Sheet_2.DOC]

**Table S1. The difference prevalence of positive Aβ and APOEε4 carrier for NSSM, SCD-SG and MCI-SG in non-cerebral infarction individuals**

| Characteristics | NSSM | SCD-SG | MCI-SG | *P* |
| --- | --- | --- | --- | --- |
| Positive Aβ, n(%) | 116(27.8) | 41(34.8) | 48(41.4) | 0.004 |
| APOEε4 carrier, n(%) | 73(17.9) | 21(20.2) | 37(32.2) | 0.002 |

NSSM include normal group, SG-A,group, SCD-A group and MCI-A group,namely except SCD-SG group and MCI-SG group.

**Table S2. OR (95% CI) for positive Aβ and APOEε4 carrier in SCD-SG and MCI-SG using normal group as a reference in non-cerebral infarction individuals**

| Characteristics | SCD-SG  OR (95% CI) | MCI-SG  OR (95% CI) |
| --- | --- | --- |
| positive Aβ |  |  |
| Normal group (reference) | 1.86(1.06-3.25)^!^ | 2.05(1.16-3.60)^!^ |
| APOE ε4 carrier |  |  |
| Normal group (reference) | 1.02(0.54-1.94) | 1.83(1.01-3.31)^!^ |

The model adjusted for age, sex, education, handgrip strength, heart disease, type 2 diabetes, osteoporosis, fracture, liver cirrhosis.

^!^ *P* < 0.05

**Table S3. OR and 95% (CI) for the associations of positive Aβ and APOE ε4 carrier with risk of SCD-SG and MCI-SG in non-cerebral infarction individuals**

| Characteristics | SCD-SG  OR (95% CI) | MCI-SG  OR (95% CI) |
| --- | --- | --- |
| positive Aβ |  |  |
| SG-A (reference) | 1.58(0.89-2.79) | 1.74(0.99-3.08) |
| SCD-A (reference) | 2.01(1.01-4.00)^!^ | 2.22(1.11- 4.42)^!^ |
| MCI-A (reference) | 0.92(0.47-1.78) | 1.01(0.53-1.94) |
| NSSM (reference) | 1.57(0.99-2.48) | 1.70(1.07-2.69)^!^ |
| APOE ε4 carrier |  |  |
| SG-A (reference) | 1.45(0.72-2.93) | 2.61(1.37-4.98)^!^ |
| SCD-A (reference) | 1.73(0.72-4.14) | 3.10(1.35-7.15)^!^ |
| MCI-A (reference) | 0.93(0.41-2.09) | 1.66(0.78-3.54) |
| NSSM (reference) | 1.20(0.69-2.11) | 2.15(1.32-3.53)^!^ |

The model adjusted for age, sex, education, handgrip strength, heart disease, type 2 diabetes, osteoporosis, fracture, liver cirrhosis.

^!^ *P* < 0.05

**Table S4. OR and 95% (CI) for the associations of positive Aβ/ APOE ε4 nocarrier, negative Aβ/APOE ε4 carrier and positive Aβ/APOE ε4 carrier with risk of SCD-SG and MCI-SG in non-cerebral infarction individuals**

| Characteristics | SCD-SG  OR (95% CI) | MCI-SG  OR (95% CI) |
| --- | --- | --- |
| Positive Aβ/APOE ε4 nocarrier vs negative Aβ/APOE ε4 nocarrier |  |  |
| Normal (reference) | 2.07(1.04-4.12)^!^ | 2.26(1.11-4.60)^!^ |
| SG-A (reference) | 1.46(0.74-2.88) | 1.59(0.79-3.20) |
| SCD-A (reference) | 1.80(0.79-4.09) | 1.96(0.85-4.55) |
| MCI-A (reference) | 0.96(0.423-2.17) | 1.05(0.46-2.37) |
| NSSM (reference) | 1.59(0.91- 2.79) | 1.67(0.95-3.05) |
| Negative Aβ/APOE ε4 carrier vs  negative Aβ/APOE ε4 nocarrier |  |  |
| Normal (reference) | 0.83(0.33-2.14) | 2.34(1.05-5.20)^!^ |
| SG-A (reference) | 0.95(0.35-2.59) | 2.67(1.14-6.28)^!^ |
| SCD-A (reference) | 0.73(0.25-2.17) | 2.05(0.78-5.38) |
| MCI-A (reference) | 0.64(0.20-2.02) | 1.78(0.64-4.92) |
| NSSM (reference) | 0.82(0.36-1.89) | 2.29(1.18-4.42) ^!^ |
| Positive Aβ/APOE ε4 carrier vs  negative Aβ/APOE ε4 nocarrier |  |  |
| Normal (reference) | 1.67(0.68-4.09) | 2.30(0.95-5.57) |
| SG-A (reference) | 2.32(0.86-6.23) | 3.19(1.20-8.46)^!^ |
| SCD-A (reference) | 12.52(1.50-104.22)^!^ | 17.23(2.07-143.14)^!^ |
| MCI-A (reference) | 1.15(0.37-3.59) | 1.58(0.53-4.73) |
| NSSM (reference) | 2.11(0.98-4.55) | 2.82(1.33-5.95) ^!^ |

Negative Aβ/APOE ε4 nocarrier, individuals with negative Aβ and APOE ε4 nocarrier; positive Aβ/APOE ε4 nocarrier, individuals with positive Aβ and APOE ε4 nocarrier; negative Aβ/APOE ε4 carrier, individuals with negative Aβ and APOE ε4 carrier; positive Aβ/APOE ε4 carrier, individuals with positive Aβ and APOE ε4 carrier.

The model adjusted for age, sex, education, handgrip strength, heart disease, type 2 diabetes, osteoporosis, fracture, liver disease.

^!^ *P* < 0.05
